# O9-8 Here, you step outside and you're into nature' Physical activity and health among first-generation Italian immigrants in Norway

**DOI:** 10.1093/eurpub/ckac094.072

**Published:** 2022-08-29

**Authors:** Giovanna Calogiuri, Laura Terragni

**Affiliations:** 1 Inland Norway University of Applied Sciences, Elverum, Norway; 2 Department of Nursing and Health Promotion, Oslo Metropolitan University, Oslo, Norway

**Keywords:** migration health, adults, older adults, environmental correlates, self-rated health

## Abstract

**Background:**

Moving to a different country often leads to changes in a person's health-related behaviours such as, among others, engagement in moderate-to-vigorous physical activity (MVPA). Italian immigrants in Norway have tripled in the past 15 years, but little is known about their lifestyle and health. The aim of this study was to examine i) the MVPA behaviour, ii) the association of MVPA with self-rated health, and iii) the perceived impact of moving among first-generation Italian immigrants in Norway.

**Methods:**

The data was retrieved from a cross-sectional survey (n = 321) and a set of in-depth interviews (n = 14) conducted within the study Mens Sana in Corpore Sano. Inclusion criteria were: age ≥18 years, living in Norway permanently, having lived in Italy at least until age 16 years. The data included information on the participants' MVPA behaviour (amount, frequency, and mode), self-rated health, and perceived impact of moving on their MVPA behaviour. Additionally, socio-demographic characteristics (sex, age, and educational level) and factors associated with the settlement process (years in Norway, contact with close friends, and national identity) were included as covariates.

**Results:**

Most of the respondents (62%) engaged in MVPA for ≥150 min/week; the mean MVPA frequency was 3.37±2.29 times/week. The most popular modes of MVPA were active transport and physical activity in natural settings. After controlling for multiple covariates, both engaging in MVPA for ≥150 min/week (β = 0.177; p = 0.002) and higher MVPA frequency (β = 0.164; p = 0.005) significantly predicted higher levels of self-rated health. While 15% of respondents perceived that moving to Norway had a negative impact on their MVPA behaviour, 50% perceived a positive impact. From the qualitative analysis, it emerged that proximity to natural environments and Norway’s culture that value outdoor recreations were important elements supporting the participants’ MVPA habits as well as their perceived health.

**Conclusions:**

In spite of the relatively high socio-economic profile of most first-generation Italian immigrants in Norway, yet their MVPA levels is somewhat lower than the Norwegian population's. The physical and cultural environment in Norway, however, appears to contribute buffering the health challenges among Italian immigrants in Norway.

